# Inhibition of stress and spontaneous respiration: Efficacy and safety of monitored anesthesia care by target-controlled infusion remifentanil in combination with dexmedetomidine in fibreoptic bronchoscopy for patients with severe tracheal stenosis

**DOI:** 10.3389/fmed.2022.972066

**Published:** 2022-10-31

**Authors:** Yi Zhou, Wei Wu, Yuanjie Zhu, Xin Lv, Jianming Liu

**Affiliations:** ^1^School of Life Sciences and Technology, Tongji University, Shanghai, China; ^2^Department of Anesthesiology, Shanghai Pulmonary Hospital, School of Medicine, Tongji University, Shanghai, China; ^3^Department of Anesthesiology, Changhai Hospital, Naval Medical University, Shanghai, China

**Keywords:** remifentanil, effect concentration, monitored anesthesia care, severe tracheal stenosis, fibreoptic bronchoscopy, spontaneous breathing

## Abstract

**Objective:**

This study aimed to determine the effective concentration of target-controlled infusion (TCI) of remifentanil used to inhibit stress during the treatment of severe tracheal stenosis with fibreoptic bronchoscopy and to evaluate the monitored anesthesia care (MAC) by remifentanil.

**Materials and methods:**

60 patients with severe tracheal stenosis who underwent fibreoptic bronchoscopy was performed. Dexmedetomidine was initially administered at a bolus dose (0.8 mcg/kg), followed by a 0.5 mcg/(kg⋅h) continuous infusion. Remifentanil was administered by TCI. The effective concentration (EC) of remifentanil was titrated by the improved sequential method, and 30 patients were included. The EC95 of remifentanil was set as the plasma target concentration to evaluate the safety of the MAC, and another 30 patients were included.

**Results:**

The half effective effect-chamber concentration of remifentanil (EC50) was 2.243 ng/ml, and the EC95 was 2.710 ng/ml. Among the 30 patients who received an EC95 of remifentanil as the target concentration, one patient was remedied by injecting propofol, the score of Ramsay sedation was three. The incidence of subclinical hypoxemia (SPO_2_ of 90–95%) was 30%, the incidence of moderate hypoxemia (SPO_2_ of 75–89%, ≤60 s) was 20 and 86.7% of patients with oxygen saturation was less than 95% returned to normal by awakening. The satisfaction score of the operator was nine, the satisfaction score of the anesthesiologist was eight, the satisfaction score of the patients was 10, the rate of patient willingness to re-accept the procedure was 93.3% and the circulation was stable during the operation.

**Conclusion:**

MAC using TCI of remifentanil with continuous pumping dexmedetomidine can effectively inhibit the stress response to fibreoptic bronchoscopy in patients with severe tracheal stenosis while maintaining spontaneous breathing. Under the anesthesia management of an experienced anesthesiologist, it provides a reference to tracheoscopic anesthesia of autonomous breathing.

**Clinical trial registration:**

[http://www.chictr.org.cn/], identifier [ChiCTR 2100043380].

## Introduction

With the development of endoscopic technology, interventional treatment *via* fibreoptic bronchoscopy has become one of the main methods of diagnosis and treatment for patients with benign and malignant tracheal stenosis, and the demand for fibreoptic bronchoscopic interventional treatment for patients with severe tracheal stenosis continues to increase (1–3). Compared with ordinary patients, severe patients tend to be anxious and exhibit obvious difficulty breathing, tachycardia, hypersecretion or expectoration of sputum, and lung infections, and they must often be placed in unnatural postures to maintain airway patency. Even if apnoea occurs, most conscious patients cannot tolerate the diagnosis and treatment of fibreoptic bronchoscopy, which could improve airway obstruction (4).

Effective and safe anesthesia management technology can inhibit the stress response to fibreoptic bronchoscopy in severe patients, reduce the occurrence of choking cough and laryngeal spasm, and reduce serious complications that may be life-threatening, such as asphyxia, massive bleeding, and malignant arrhythmia (5, 6). There is no compressed air in the respiratory endoscopy center of our hospital, so, laryngeal mask general anesthesia can only provide pure oxygen during mechanical ventilation, which is more likely to cause airway fire and damage during laser cauterization. MAC with spontaneous respiration has significant advantages over local anesthesia and laryngeal mask general anesthesia (7–11). However, there are few studies about MAC on anesthesia management techniques for such severe patients at home and abroad. In our study, on the basis of sedation with dexmedetomidine, the effective concentration of remifentanil for inhibiting the stress response was titrated through a modified sequential method during fibreoptic bronchoscopy of patients with severe tracheal stenosis, and the safety of the MAC regimen with remifentanil was evaluated.

## Materials and methods

### Study design and population

This prospective interventional study was conducted at the respiratory endoscopy center of Shanghai Pulmonary Hospital affiliated with Tongji University from February 2021 to May 2021. The study was approved by the Ethics Committee of Shanghai Pulmonary Hospital, Tongji University, China (K19-122) and registered in the Chinese Trial Registry (12/02/2021, ChiCTR2100043380). All patients enrolled in this study received written informed consent.

Sixty patients who received fibreoptic bronchoscopy treatment were included, all of whom were diagnosed with severe tracheal stenosis for the first time. The effective concentration of remifentanil was titrated by the improved sequential method in 30 patients, the safety of the MAC protocol was evaluated using EC95 as the plasma target concentration in 30 patients, and the inclusion of patients was completed by LJM. The inclusion criteria included patients with severe tracheal stenosis (the reduced area of the tracheal cavity was more than 50%) who wanted to be treated by fibreoptic bronchoscopy, were aged 18 ∼ 65 years, and had ASA I–III status. The exclusion criteria were as follows: abnormal nasal anatomy, severe coagulation dysfunction, severe hepatic and renal dysfunction, history of abnormal recovery from surgical anesthesia, chronic opioid treatment, substance abuse or drug use, pregnancy, history of allergy to related drugs, and no informed consent. Included patients were later excluded if general anesthesia by laryngeal-mask or endotracheal intubation was required for the operation or the operation was over 60 min in duration.

### Monitored anesthesia care protocol

All patients fasted for 8 h, and water was forbidden for 4 h preoperatively. In the anesthesia preparation area, 0.03 mg/kg midazolam (Midazolam^®^, Nhwa, China) was given intravenously to relieve pre-procedural anxiety. Electrocardiogram (ECG), heart rate (HR), pulse oxygen saturation (SpO_2_) and mean arterial pressure (MAP), and respiratory rate (RR) were monitored regularly after patient entry into the operating room. Oxygen inhalation through a nasal catheter (2 L/min) was performed. A simple mask breathing apparatus and anesthesia machine were used as a standby. Dexmedetomidine (0.8 mcg/kg, Dexmedetomidine^®^, Yangtze River, China) was administered within 10 min using a Fresenius DPS workstation, and plasma target-controlled infusion (TCI) of remifentanil (Remifentanil^®^, Yichang Humanwell, China) was completed within 5 min. When the effector chamber concentration reached the target concentration, a nasopharyngeal airway (No. 6/7, Medis, UK) was placed, and oxygen was given by an anesthesia machine (6 L/min) with an adjustable pressure-limiting (APL) valve setting of 30 cm H_2_O. There was no compressed air in the respiratory endoscopy center of our hospital and only pure oxygen was provided. Four milliliters of 1% lidocaine (Lidocaine^®^, CSPC, China) was injected through the nasopharyngeal airway for topical anesthesia, and then fibreoptic bronchoscopy was started. When the fibreoptic bronchoscope (BF-1T260/6C260, Olympus, Japan) was placed, 4 ml of 1% lidocaine was injected through the bronchoscopic tube into the acoustic gateway and subglottis for topical anesthesia. Intraoperative dexmedetomidine was pumped continuously at 0.5 mcg/(kg⋅h). The effective concentration of remifentanil was titrated by a modified sequential method. The plasma target concentration of remifentanil in the first patient was 2.5 ng/ml, and the difference between adjacent targets was 0.5 ng/ml. After three cycles of negative and positive reactions, the difference in adjacent target concentrations was changed to 0.2 ng/ml. The stress response was defined as positive if the change in HR or MAP exceeded 15% of the baseline or a choking cough affected the operation. Intravenous injection of 10∼20 mg propofol was used as a remedy and was used repeatedly if necessary. After obtaining the EC95 of remifentanil, the plasma target concentration was set to EC95 to evaluate the perioperative safety of the MAC during the operation.

Related events and their management: (1) definition of hypoxemia: SpO_2_ < 90% at any time. The severity of hypoxemia was classified as follows: subclinical hypoxemia (SPO_2_ of 90–95%), moderate hypoxemia (SPO_2_ of 75–89%, ≤60 s), and severe hypoxemia (SpO_2_ < 90% for >60 s or SpO_2_ < 75% at any time). The treatment process for hypoxemia was as follows: stimulation and awakening, increasing the oxygen flow (10 L/min), supporting the lower jaw, mask-assisted ventilation, and mechanical ventilation with a laryngeal mask. (2) hypotension: for MAP < 80% of baseline or 60 mmHg, if necessary, an intravenous injection of norepinephrine (25∼100 μg/time) was used to maintain the blood pressure and was repeated when needed. (3) bradycardia: HR < 50 bmp, with administration of atropine as appropriate; if arrhythmia occurred, vasoactive drugs were administered by the anesthesiologist based on his or her clinical judgment.

Cough score is 0–2, 0: no choking cough, 1: Choking cough does not affect operation, 2: choking cough affects operation. The criteria for the Ramsay sedation score are 1–6, 1: Not quiet and irritable, 2: Quiet cooperation, 3: Sleepy, can follow instructions, 4: Sleep state, but can wake up, 5: Sleep state, response to strong stimuli, but lags in response, 6: Deep sleep state, the call is not to wake up. Score 2–4 are satisfactory with sedation and 5–6 have excessive sedation.

### Outcome measures

The primary outcome measures were the cough score and the incidence and severity of hypoxemia. The secondary outcomes included recovery time; dosage of propofol; Ramsay score; arterial blood gas analysis before and after the operation; hemodynamic changes; the tolerance score for nasopharyngeal airway placement; satisfaction scores of the operator, anesthesiologist and patient; throat pain and epistaxis at 30 min after the end of the operation; throat pain; patients’ scores on operation recall and willingness to receive treatment again at 24 h; and related adverse events such as post-operative pruritus, nausea and vomiting, bleeding, hemoptysis requiring invasive re-treatment, pneumothorax, etc.

### Statistical analysis

All statistical analyses were performed using SPSS 26.0. Continuous variables are presented as the mean [standard deviation (SD)] or median [interquartile range, (IQR)]. The non-normally distributed data are presented as the median [IQR]. Categorical variables are presented as counts (%). Continuous variables were compared using the Mann-Whitney *U* test or *t*-test. EC95, EC50, the standard error and the logarithm value of the 95% confidence interval (CI) of remifentanil were calculated by the formula of the sequential method (12). The sample size of the effective concentration titrated by the improved sequential method is not clearly defined. A sample size of 20–40 has been used in general studies (12). In the present study, the sample size of the effective concentration titrated by remifentanil was 30. The safety of 30 patients was also observed. Two-sided *p*-values <0.05 were considered significant.

## Results

### Patients

**FIGURE 1:**
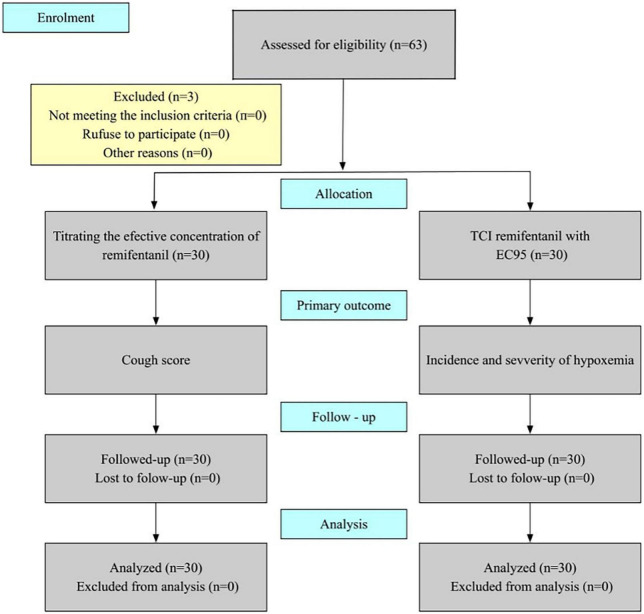
Consort flow diagram.

All 63 patients enrolled in the study; three patients were excluded because the operation time was more than 60 min, 60 patients were eligible for the data analysis, and none were discontinued due to safety concerns. The flow chart is shown in Figure 1. Demographic and operation-related data for all 60 patients are shown in Table 1.

**TABLE 1:** Characteristics of patients in this study (*n* = 60).

Item	
Age, mean ± SD, years	48.6 ± 12.8
Male, *n* (%)	17 (56.7)
Height, mean ± SD, m	1.7 ± 0.1
Weight, mean ± SD, kg	64.8 ± 9.3
BMI, mean ± SD, kg/m^2^	23.8 ± 2.6
ASA 1/2/3, *n* (%)	0(0)/42(70.0)/18(30.0)
Classification of airway stenosis	
2/3/4, *n* (%)	30(50)/24(40)/6(10)
Indications for bronchoscopy, *n* (%)	
Malignant tumor of trachea	30 (50.0)
Benign tumor of trachea	14 (23.3)
Stenosis after tracheotomy	6 (10.0)
Tracheomalacia	4 (6.7)
Other	6 (10.0)
Diagnostic interventions, *n* (%)	
Tumor removal	16 (26.7)
Tumor cauterization and cryopreservation	22 (36.7)
Stent placement	18 (30.0)
Balloon dilatation	4 (6.7)
Pre−bronchoscopic respiratory parameters	
SpO_2_, median [IQR],%	96 [95–97]
RR, mean ± SD, per min	16 ± 2
PaO_2_ [IQR], mmHg	82.0 [77.0–86.5]
PaCO_2_ [IQR], mmHg	41.5 [40.3–43.7]
Pre−bronchoscopic hemodynamic parameters	
MAP, median, mmHg	96.5 ± 6.4
HR, mean ± SD, beat per min	89.2 ± 18.1

SpO_2_, pulse oxygen saturation; IQR, interquartile range; RR, respiratory rate; SD, standard deviation; PaO_2_, arterial oxygen partial pressure; PaCO_2_, arterial carbon dioxide partial pressure; MAP, mean arterial pressure; HR, heart rate.

### Effective concentration of remifentanil

**FIGURE 2:**
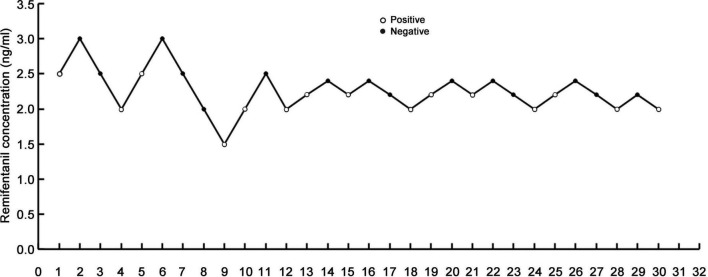
Stress response of patients to fiberoptic bronchoscopy.

Figure 2 shows that the stress response of 30 patients with severe tracheal stenosis during fibreoptic bronchoscopy treatment was treated with remifentanil at different blood concentrations using the modified sequential method. The half effective effect-chamber concentration of remifentanil (EC50) was 2.243 ng/ml (95% CI, 2.061–2.446 ng/ml), and the EC95 was 2.710 ng/ml (95% CI, 2.471–4.473 ng/ml), as shown in Table 2.

**TABLE 2:** Confidence interval of remifentanil effective concentration.

Confidence limit	95% Confidence limit		
Probability	Estimate	Lower limit	Upper limit
0.01	1.717	0.858	1.937
0.05	1.857	1.13	2.035
0.1	1.936	1.308	2.092
0.15	1.991	1.441	2.134
0.2	2.036	1.555	2.17
0.25	2.076	1.657	2.205
0.3	2.112	1.752	2.241
0.35	2.146	1.84	2.28
0.4	2.179	1.921	2.325
0.45	2.211	1.996	2.38
0.5	2.243	2.061	2.446
0.55	2.276	2.117	2.528
0.6	2.309	2.166	2.628
0.65	2.345	2.207	2.746
0.7	2.383	2.245	2.885
0.75	2.424	2.281	3.05
0.8	2.471	2.317	3.251
0.85	2.527	2.357	3.508
0.9	2.599	2.404	3.866
0.95	2.71	2.471	4.473
0.99	2.931	2.596	5.895

### Information related to surgery, hypoxemia and related adverse reactions

**FIGURE 3:**
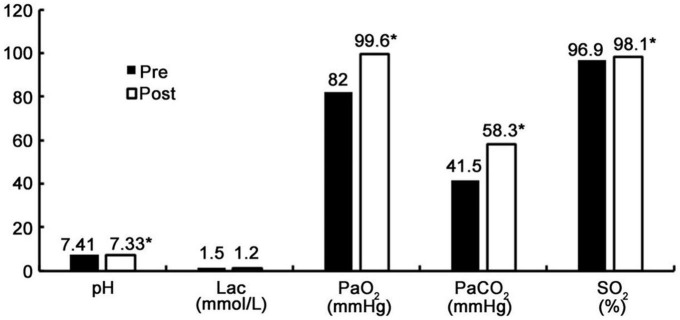
Results of arterial blood gas analysis before and after procedure. *P < 0.05.

For 30 patients with EC95 TCI of remifentanil, hemodynamics were stable at each time point during the operation, as shown in Table 3. One case with remedy by 30 mg propofol was completed. The tolerance score for nasopharyngeal airway placement, Ramsay sedation score, cough score and satisfaction score are shown in Table 4. The incidence of subclinical hypoxemia was 30%, the incidence of moderate hypoxemia was 20%, and the incidence of severe hypoxemia was 0%. Among all patients with oxygen saturation was less than 95%, 86.7% were restored to normal by awakening, one was restored to normal by mask-assisted ventilation, and another was restored to normal by laryngeal-mask mechanical ventilation (Table 5). The analysis of arterial blood gas before and after the operation is shown in Figure 3. Other sedation-related adverse reactions are shown in Table 6. There were cases of increased heart rate and blood pressure, none of which exceeded 20% of the baseline value. Adverse reactions related to oxygen delivery, including throat pain 30 min and 24 h after the operation, are shown in Table 7.

**TABLE 3:** Hemodynamic changes (HR, beats/min; MAP, mmHg; *n* = 30).

Item	T_0_	T_1_	T_2_	T_3_	T_4_	T_5_
HR	96.4 ± 18.7	80.8 ± 12.9	91.4 ± 17.3	85.3 ± 12.1	80.4 ± 11.1	78.1 ± 8.9
MAP	101.7 ± 10.0	89.9 ± 9.4	99.8 ± 12.2	93.1 ± 8.1	85.9 ± 6.1	85.4 ± 7.3

T_0_, before procedure; T_1_, procedure; T_2_, 5 min after procedure; T_3_, 10 min after procedure; T_4_, 15 min after procedure; T_5_, end of procedure.

**TABLE 4:** Procedure data, propofol dosage and satisfaction (*n* = 30).

Characteristic	
Procedure time, min	25.7 ± 8.1
Recovery time, min [IQR]	2 [1.0–2.3]
Sedation score [IQR]	3 [3–4]
Nasopharynx airway tolerance score [IQR]	2 [2–3]
Cough score [IQR]	1 [1–1]
SpO_2_, median [IQR],%	99 [95–100]
RR, mean ± SD, per min	10 ± 2
Propofol dose, mg, *n* (%)	30, 1 (3.3)
Patient satisfaction score [IQR]	10 [10–10]
Bronchoscopist satisfaction score [IQR]	9 [9–10]
Anesthesiologist satisfaction score [IQR]	8 [8–8.5]
24-hour patient recall score for operation [IQR]	1 [0–1]
Patients’ willingness to accept the operation again, yes, *n* (%)	28 (93.3)

IQR, interquartile range; SpO_2_, pulse oxygen saturation; RR, respiratory rate; SD, standard deviation.

**TABLE 5:** Incidence of hypoxemia and need for airway assistance (*n* = 30).

Characteristic	
Respiratory depression, *n* (%)	15 (50.0)
Subclinical respiratory depression	9 (30.0)
Moderate hypoxemia	6 (20.0)
Severe hypoxemia	0 (0)
Need for airway assistance	15 (50.0)
Stimulation	13 (43.3)
Increasing oxygen delivery	0 (0)
Jaw thrust	0 (0)
Mask ventilation	1 (3.3)
Mechanical ventilation	1 (3.3)

**TABLE 6:** Other adverse events related to the sedation (*n* = 30).

Item	
Adverse even, *n* (%)	7 (23.3)
Minimal risk	0 (0)
Nausea/Vomiting	0 (0)
Muscle rigidity, myoclonus	0 (0)
Agitation during recovery	0 (0)
Prolonged recovery	0 (0)
Minor risk	7 (23.3)
Airway obstruction	0 (0)
Failed sedation	0 (0)
Allergic reaction without anaphylaxis	0 (0)
Bradycardia	0 (0)
Tachycardia	2 (6.7)
Hypotension	0 (0)
Hypertension	5 (16.7)
Sentinel risk	0 (0)
Cardiovascular collapse/shock	0 (0)
Cardiac arrest/absent pulse	0 (0)

**TABLE 7:** Adverse events related to oxygen delivery system (*n* = 30).

Adverse event	
30 min after procedure	5 (16.7)
Sore throat	5 (16.7)
Epistaxis	0 (0)
Dry mouth	0 (0)
24 h after procedure	2 (6.7)
Throat pain	2 (6.7)
Dry mouth	0 (0)

## Discussion

Through the improved sequential method, we concluded that the EC95 for remifentanil inhibition of the stress response in fibreoptic bronchoscopy for patients with severe tracheal stenosis was 2.710 ng/ml (95% CI, 2.471–4.473 ng/ml) based on dexmedetomidine sedation and that the EC50 was 2.243 ng/ml (95% CI, 2.061–2.446 ng/ml). For all 30 patients, spontaneous breathing was retained during the diagnosis and treatment period, which improved the perioperative safety of severe patients and provided an effective and a new concept anesthesia scheme for patients with severe tracheal stenosis who were undergoing fibreoptic bronchoscopy.

With the development of fibreoptic bronchoscopy technology, it has been widely used in clinical practice, and more than 5,00,000 bronchoscopy procedures are performed in the United States every year (13). A large number of clinical studies have confirmed that the stress response cannot be effectively suppressed under only local anesthesia, which may lead to choking cough or laryngeal spasm resulting in a decrease in PaO_2_, aggravating the patient’s dyspnoea, interrupting the operation, and even causing serious life-threatening complications such as asphyxia, massive bleeding, malignant arrhythmia, and so on. Except for patients with obvious contraindications, the guidelines recommend routine sedation for all patients undergoing fibreoptic bronchoscopy (10, 14, 15). The application of sedative medicine during fibreoptic bronchoscopy can effectively improve the patient’s tolerance, reduce the choking cough during the operation and increase the patient’s willingness to revisit the diagnosis and treatment without significantly increasing the related complications (15–17). Compared with general patients, patients with severe stenosis are less tolerant to fibreoptic bronchoscopic intervention, which may improve airway obstruction. Therefore, it is a great challenge for anesthesiologists to provide anesthesia management for patients with severe tracheal stenosis through fibreoptic bronchoscopy, and there is currently no recognized standardized anesthesia management plan here or abroad (15, 18). There are unique challenges to anesthesiologists sharing the airway with the operator. The implementation of sedation and anesthesia reduces risk, improves the comfort of patients and operators and increases the continuity and success of the procedure. In 2009, a study in China showed that two out of 58 hospitals routinely used general anesthesia-assisted or controlled ventilation through laryngeal masks to complete such endoscopic diagnosis and treatment (19). However, anesthesia tolerated with the laryngeal mask may result in significant circulation inhibition and longer recovery time. In recent years, from the perspective of protecting the health and safety of medical personnel and patients and reducing costs, the number of stent placement for tracheal stenosis under X-ray fluoroscopy has gradually decreased. If the stent placement of airway stenosis (especially Y-shaped stent) is not positioned under X-ray, it is necessary to use the fiberoptic bronchoscope to accurately position the stent during the release process. In this case, when the laryngeal mask is used, the laryngeal mask space cannot accommodate the releaser and fiberoptic bronchoscope at the same time, and the arc shape of the laryngeal mask itself is not conducive to the operation of the endoscopist. Our experience shows that in this case, repeated airway operations can easily lead to laryngeal mask displacement. Laryngeal mask is a ventilation device on the glottis, when without compressed air and the ventilation effect is good, the airway is equivalent to a closed pure oxygen state after mechanical ventilation. However, the nasopharynx airway is placed in the nasopharynx, even if the anesthesia machine is connected to give oxygen, it only increases the local oxygen concentration in the nasopharynx, and the oxygen concentration in the airway is less than 100%. Because there is no compressed air in the respiratory endoscopy center of our hospital, laryngeal mask general anesthesia can only provide pure oxygenin. In this case, compared with nasopharynx airway, laryngeal mask ventilation has higher oxygen concentration in the airway, which is more likely to cause airway fire and damage during laser cauterization. It may even be necessary to suspend ventilation during operation. The main difference between laryngeal mask general anesthesia and MAC is whether to keep autonomous breathing. MAC can reduce the amount of anesthetic drugs, reduce medical expenses, and better meet the turnover of outpatient surgery patients. It has less impact on respiratory function and is more suitable for patients with respiratory dysfunction. However, the potential anesthetic risk may be higher and senior anesthesiologists are required to monitor during the whole process of operation. Of course, timely detection of problems and early intervention should reduce the risk and improve the safety. Compared with local and general anesthesia laryngeal mask, the MAC with autonomous breathing provided by the nasopharyngeal airway for oxygen has obvious advantages (19).

Sequential methods, also known as up-down methods or step-down methods, are simpler and more effective methods to study the effective concentration of drugs. The advantage of the sequential method is that it can make full use of the data provided by fewer cases and obtain results quickly and accurately, which can reduce the number of trial cases by 30 ∼ 40%. Remifentanil has a quick onset and rapid elimination, TCI makes its dose accurate and easy to adjust, and the inhibition of cardiovascular responses caused by stress can be quickly determined, which is suitable for sequential study (20, 21). EC50 refers to half of the subjects at a particular reaction dose and can be sensitive in reflecting changes in the drug concentration and effect. EC95 refers to the effective concentration for 95% of subjects with a specific reaction. The EC50 study concentration-response relationship of a drug is more sensitive and accurate than the EC95; however, the effectiveness of the EC95 is higher, and drug-related adverse reactions may be increased because of the higher drug concentration. In the second part of this study, the EC95 of remifentanil was used to evaluate patients’ hypoxemia and other related adverse reactions, and its safety could be investigated better.

Our study showed that the incidence of oxygen saturation was less than 95% was 50% (15/30), that of subclinical hypoxemia was 30% (9/30), that of moderate hypoxemia was 20% (6/30), and that of severe hypoxemia was 0% (0/30) among 30 patients with TCI with EC95 of remifentanil. Respiratory depression can be manifested as the decrease of respiratory rate, the decrease of oxygen saturation, and the increase of end expiratory carbon dioxide. This study uses the decrease of oxygen saturation as the main observation index. During the research design, it is considered that the accuracy of oxygen saturation is high, the error is small, and the operation is simple. A total of 86.7% (13/15) of the patients with oxygen saturation was less than 95% returned to normal by wakening, one patient returned to normal by face-mask-assisted ventilation, and another patient returned to normal by laryngeal-mask mechanical ventilation. The patient with laryngeal-mask mechanical ventilation was 65 years old, weighed 46 kg, and had a height of 175 cm, a BMI of 15, hypertension, diabetes, and 75% airway stenosis. The Ramsay sedation score was five, the lowest SpO_2_ was 85%, and SpO_2_ became 100% by mask-assisted ventilation; however, breath was still not recovered. The operation was successfully completed through mechanical ventilation with the laryngeal mask, and the changes in MAP and HR did not exceed 10% of the baseline. The patient awakened 8 min after the operation, and no adverse reactions were found during the 24 h follow-up. This patient was analyzed as a frail patient with hypertension and diabetes accompanied by advanced age and low body weight. The EC95 was 2.710 ng/ml (95% CI, 2.471–4.473 ng/ml) for this patient, and the depth of anesthesia may have been too deep, excessive sedation, leading to moderate hypoxemia. In the study, subclinical hypoxemia refers to oxygen saturation greater than 90 and less than 95, and patients will not suffer from hypoxia. And Moderate hypoxemia means that the oxygen saturation is greater than or equal to 75, less than 90 s. In this study, the amount of dexmedetomidine and the target concentration of remifentanil are constant, and the anesthesia is too deep for some patients, which is also one of the main reasons why 20% of patients have moderate hypoxemia. The anesthesia dosage can also be adjusted based on the EC50 of remifentanil. In the actual clinical operation, each patient is individualized. However, the dosages of dexmedetomidine and remifentanil in this study are the reference dosages of clinical anesthesia. In clinical practice, anesthesiologists need to adjust them in real time according to the reaction of patients to drugs and the operation steps.

In the study, the blood gas analysis value is obtained by a single arterial blood collection at two time points before anesthesia and at the end of the operation.

The results show that the SpO_2%_ median (IQR) before and after the operation was [96 (95–97) and 99 (95–100), *P* < 0.05], PaO_2_ (mmHg) median (IQR) values were [82.0 (77.0–86.5) and 99.6 (85.0–145.2), *P* < 0.05]. The increase in SpO_2_ and PaO_2_ during the operation may have been related to the use of the nasopharyngeal airway (22) (No. 6/7, Medis, UK); oxygen was delivered by the anesthesia machine (6 L/min), and the APL valve was set to 30 cm HO_2_. This special nasopharyngeal airway can be connected with an anesthesia machine to supply oxygen, providing a higher concentration and more effective oxygen therapy than nasal catheters. At the same time, changes in end-expiratory carbon dioxide and respiratory rate can be continuously monitored to detect respiratory depression as early as possible and even provide an early warning before the occurrence of decreased SpO_2_ to reduce the risk of clinical hypoxemic events. In the future, relevant randomized controlled studies can be designed to obtain evidence-based medicine evidence. The PaCO_2_ (mmHg) median (IQR) before and after the operation was [41.5 (40.3–43.7) and 58.3 (50.7–63.0), respectively, (*P* < 0.05)], the pH median (IQR) values were [7.41 (7.39–7.43) and 7.33 (7.28–7.36), *P* < 0.05], and the Lac (mmol/L) median (IQR) values were [1.50 (1.20–1.80) and 1.20 (1.00–1.80), *P* > 0.05]. Respiratory depression that occurred during the operation led to an increase in PaCO_2_, but all of these values were <70 mmHg, which was within the range of permissible hypercapnia. The absolute level of PaCO_2_ and the permissible degree of acidosis is debated, which is the concern of alveolar derecruitment and possible worsening of ventilation-perfusion mismatching (23). Permissive hypercapnia has not been widely implemented to near its physiologic limits (PaCO_2_ up to 80 mmHg, arterial pH down to 7.20) (23). In current practice, mean maximum PaCO_2_ and pH associated with permissive hypercapnia are around 67 mmHg and 7.2, respectively, (24). It was reported that hypercapnia can be well tolerated as long as tissue perfusion and oxygenation are preserved and there are no contraindications (25). But hypercapnia may cause cardiovascular and cerebrovascular problems and acid-base imbalance. The changes in pH and Lac were clinically within acceptable ranges, and the patients’ circulation was stable. There was no special treatment in clinical practice. One patient was treated with propofol because performance of the operation was affected by choking cough; 30 mg of propofol was injected twice intravenously in order to complete the operation. The hemodynamics of all patients were stable at all time points during the operation, and no vasoactive drugs were used, indicating that this MAC can effectively inhibit such stress without affecting circulatory stability. The median tolerance score for nasopharyngeal airway placement was two, the median Ramsay sedation score was three, the median cough score was one, the median operator-physician satisfaction score was nine, the median anesthesiologist satisfaction score was eight, the median patient satisfaction score was 10, the median patient recall score for 24 h was one, and the willingness of patients to accept the procedure again was 93.3%. The results show that the MAC scheme of this study provides a comfortable process of diagnosis and treatment for patients and makes the operator more comfortable completing the operation. However, the satisfaction of anesthesiologists is not as high as that of operators and patients, which may be related to the continual focus of anesthesiologists on the patients’ breathing status. Anesthesiologists have to spend more effort completing anesthesia-related tasks. It is worth noting that the MAC scheme in this study is secure under the anesthesia management of anesthesiologist with rich clinical experience. This MAC puts forward high requirements for anesthesiologists and brings a lot of benefits to patients and surgeons. In clinical work, there is no best anesthesia method, only the most appropriate anesthesia method. Other sedation-related adverse effects included an increasing heart rate and blood pressure, none of which exceeded 20% of baseline. Thirty minutes after the operation, five patients (16.7%) had laryngopharyngeal pain, with VAS < 3. The MAC technique of fibreoptic bronchoscopy is complicated, poses a high risk of respiratory depression and exacts a high demand from anesthesiologists. Studies have shown that 50% of bronchoscope-related adverse events are related to sedation or (and) anesthesia implementation, which is the main reason for the low rates of such surgical sedation and anesthesia procedures in China (26–28). In the process of MAC, sedation and inhibition of the airway response are mainly achieved by drugs. At present, there is no single drug that can perfectly achieve this purpose; consequently, the combined application of local anesthesia, sedatives and opioids is clinically selected for MAC. In the UK, benzodiazepines are reported to be the most commonly used drugs (63%), followed by opioids (14%) and benzodiazepines combined with opioids (12%). The latest Australian and New Zealand censuses showed 53% use of midazolam and fentanyl. In China, benzodiazepines and/or opioids for sedation were found to be used in 44% of 58 hospitals (28–30). Remifentanil can effectively inhibit choking cough, is also the mainstream clinical and ultra-short-acting opioid, is effectively and rapidly metabolized, and can better and more efficiently meet the demands of clinical operation. However, the literature has reported that chest wall rigidity and bradycardia often occur (31, 32). This study did not observe associated adverse events, which may have been related to the accurate quantitation of TCI. Minimal anesthetic drugs were used to inhibit stress and to reduce adverse reactions, while 5 min was set to reach the plasma target concentration. Dexmedetomidine (33) is a new sedative and analgesic drug that does not easily cause respiratory depression and has obvious sedative effects. It can cause arousal sedation or cooperative sedation, is similar to normal sleep, and can reduce the dosage of opioid analgesics and adverse reactions. Therefore, in this study, the combination of remifentanil and dexmedetomidine reduced the incidence of respiratory depression and other drug-related adverse reactions.

The shortcomings of this study are as follows: it was a prospective interventional study, not a randomized controlled study, and it did not perform comparisons with other MAC regimens. Unfortunately, our study did not carry out invasive arterial monitoring, and did not continuously monitor the changes of oxygen partial pressure and carbon dioxide. The MAC has advantages, but there is a risk of hypoxemia or hypercapnia. However, we believe that the risk will be reduced significantly and improve patient safety under close anesthesia management.

## Conclusion

In summary, our study demonstrates that the MAC of remifentanil with spontaneous breathing provides a satisfactory sedative and analgesic effect for patients with severe tracheal stenosis during fibreoptic bronchoscopy. The EC95 of remifentanil for inhibiting the stress response of the operation was 2.710 ng/ml (95% CI, 2.471–4.473 ng/ml) and the EC50 was 2.243 ng/ml (95% CI, 2.061–2.446 ng/ml). The stress of the remaining patients was effectively suppressed, and the satisfaction of both the operator and the patient was high. Comfortable medical treatment of the patients was realized under the MAC. However, there may be a risk of hypoxemia or hypercapnia, anesthesiologists need to closely monitor the changes of patient’s respiratory and deal with them in time. The study provided a possible choice for the anesthesia management of such high-risk special patients and explored personalized anesthesia management schemes.

## Data availability statement

The raw data supporting the conclusions of this article will be made available by the authors, without undue reservation.

## Ethics statement

The studies involving human participants were reviewed and approved by Tongji University Affiliated Shanghai Pulmonary Hospital. The patients/participants provided their written informed consent to participate in this study.

## Author contributions

YiZ was responsible for the actual development of the research, designed the work, obtained and analyzed the data, and wrote and revised the manuscript. WW, YuZ, and XL acquired and analyzed data and revised the manuscript. JL was general designer of the research, has made contributions to the concept and design of the work, confirmed the reply of all reviewers, reviewed and revised the manuscript, and confirmed the final version of the manuscript. All authors contributed to the article and approved the submitted version.
